# Domestic Summary

**Published:** 1840-04-18

**Authors:** 


### DOMESTIC SUMMARY.

*Accidents from Arsenic.* By JOHN MILLMAN. The frequent accidents which occur from the great facility of obtaining arsenic for destroying rats, and also for criminal purposes, render it incumbent upon all of those who vend the article to adopt such measures as would enable any one to detect its presence in the ordinary articles of food, &c., it might be mixed with.

In France, where the legal restrictions imposed upon the sale of poisons are infinitely more strict than we can ever expect them to be in this country, we find gentlemen of our profession deeply interested in this subject, and recently Mr. Grimeau's plan has been submitted to the consideration of the Pharmaceutical Society of Paris. Mr. G. proposes to colour all the arsenious acid, sold in commerce under different denominations, with a mixture of sulphate of iron and cyanuret of potassium; the minute proportions of a hundredth part of each of those substances would suffice to impart such striking colours to the various articles of food, &c. which it might have been mixed with, as would at once serve as a caution to the least experienced eye. In cases where the above preparations had been given with criminal intent, judicial investigations would be much aided, inasmuch as the colouring effect would last several days.

Should arsenic be needed to mix with lime for seeding, "chaulage," Mr. G. recommends, in addition to the above, spirits of turpentine; and for medicinal and veterinary purposes, oil of lavender, in sufficient quantities to give a very strong odour to the mixture.

How far Mr. Grimeau's plan would tend to diminish the evil under consideration, is a matter well worthy our deep solicitude. Perhaps it may be in our power, by the adoption of some such precautionary measures, to supply the want of legislative action upon so important a subject.

This is also a proper occasion to remark upon the distressing occurrences occasioned by laudanum, and the stronger preparations of opium, which are so heedlessly placed within the reach of all, without any safeguard. Could not some regulations be adopted with regard to these also? Such as selling those preparations in vials, so strikingly different from those in common use as to secure the attention of the most negligent. Here, also, it must be admitted, a reform is needed: how often has laudanum been given for paregoric, although the vial has been labelled as the law directs?

*American Journal of Pharmacy.*

